# Transient and permanent hydrocephalus following resection of brain metastases located in the posterior fossa: incidence, risk factors and the necessity of perioperative external ventricular drainage placement

**DOI:** 10.1007/s11060-024-04890-1

**Published:** 2024-11-28

**Authors:** Ehab Shabo, Anna-Laura Potthoff, Thomas Zeyen, Julian P. Layer, Stefan Ehrentraut, Jasmin Scorzin, Felix Lehmann, Nils Christian Lehnen, Mohammed Banat, Johannes Weller, Florian Gessler, Daniel Paech, Motaz Hamed, Valeri Borger, Alexander Radbruch, Ulrich Herrlinger, Leonie Weinhold, Hartmut Vatter, Matthias Schneider

**Affiliations:** 1 Department of Neurosurgery, University Hospital Bonn, Venusberg-Campus 1, 53127 Bonn, Germany; 2 Department of Neurooncology, Center of Neurology, University Hospital Bonn, Bonn, Germany; 3 Department of Radiation Oncology, University Hospital Bonn, University of Bonn, Bonn, Germany; 4 Institute of Experimental Oncology, University Hospital Bonn, University of Bonn, Bonn, Germany; 5 Department of Anesthesiology and Intensive Care Medicine, University Hospital Bonn, Bonn, Germany; 6 Department of Neuroradiology, University Hospital Bonn, Bonn, Germany; 7 Department of Neurosurgery, Rostock University Medical Center, Rostock, Germany; 8 Department of Medical Biometry, Informatics and Epidemiology, University Hospital Bonn, 53127 Bonn, Germany

**Keywords:** Postoperative hydrocephalus, Posterior fossa metastasis, External ventricular drainage

## Abstract

**Purpose:**

Prophylactic insertion of an external ventricular drainage (EVD) prior to the resection of posterior fossa metastases (PFMs) is a common approach to address postoperative transient and permanent hydrocephalus. However, predicting surgery-related hydrocephalus in the preoperative phase continues to be a challenge. This study aims to analyze the incidence, preoperatively collectable risk factors and necessity of perioperative external ventricular drainage placement after posterior fossa metastasis surgery.

**Methods:**

All patients undergoing surgery for PFMs at the authors’ neuro-oncological center between 2015 and 2021 were identified and assessed for postoperative hydrocephalus occurrence. Tumour volume, edema volume, and 4th ventricle volume were assessed on preoperative magnetic resonance imaging scans using the IntelliSpace Portal 5.0. A multivariable logistic regression analysis was performed to identify possible predictors for postoperative hydrocephalus occurrence.

**Results:**

Postoperative hydrocephalus occurred in 14 of the 130 identified PFM patients (11%). Multivariable analysis and receiver operating characteristic (ROC) analysis revealed a 4th -ventricle-to-tumor-volume ratio ≤ 0.02 (OR 33.1, 95% CI 3.8-284.3, *p* = 0.001), an edema-to- tumor-volume ratio ≤ 0.85 (OR 10.6, 95% CI 2.4–47.4, *p* = 0.002), an imaging-morphological contact to the 4th ventricle (OR 5, 95% CI 1.4–18, *p* = 0.013), and multiple intracranial metastases (OR 2.4, 95% CI 1-5.9, *p* = 0.045) as independent predictors for surgery-related postoperative hydrocephalus occurrence.

**Conclusion:**

The present study identifies preoperatively detectable risk factors for the occurrence of postoperative hydrocephalus following surgery for PFMs. These findings may provide guidance in clinical decision-making regarding prophylactic EVD placement.

## Introduction

Metastatic brain lesions are the prevailing adult brain neoplasms detected in up to 40% of individuals with advanced-stage cancer [1, 2]. The incidence of brain metastases (BMs) is increasing due to enhanced imaging techniques and improved survival rates in various cancer types. BMs in the posterior fossa account for approximately 15–25% of intracranial BMs [3, 4]. Depending on clinical presentation and anatomical risk factors such as tumor volume, edema or ventricular compression, these lesions may require surgical resection followed by stereotactic radiotherapy [5].

The placement of an external ventricular drainage (EVD) is a well-established surgical procedure utilized to manage intracranial hypertension and is commonly employed during the removal of posterior fossa tumors that helps brain relaxation during surgery and provides better surgical corridor for tumor resection [6–12]. In cases of acute obstructive hydrocephalus in the early postoperative phase following resection, EVD placement can be a critical life-saving intervention. However, this additional surgical procedure may also increase the risk of complications, such as infection or bleeding, and may lead to a higher cumulative risk of wound-related issues due to the presence of two wounds instead of one—the first from the resection surgery and the second from the EVD insertion. This possibility underscores the potential need for careful, individualized assessment. Predicting the incidence of postoperative hydrocephalus prior to surgical intervention remains challenging and up to now, there is no unanimous agreement regarding the indication of prophylactic perioperative EVD placement in patients undergoing the removal of posterior fossa tumors [7, 10, 11]. Therefore, the decision on prophylactic EVD placement relies on internal institutional standards and the personal experience and choice of the attending surgeon.

The primary objective of this study was to identify predictors that can preoperatively assess the risk of postoperative hydrocephalus at a stage when key prognostic factors of metastatic cancer have not yet been established. This is particularly important, as the neurosurgeon must decide on neurosurgical resection at this point, making the decision reflective of real-world clinical practice.

## Methods

### Patient selection and inclusion criteria

All patients who underwent resective surgery for posterior fossa metastases (PFMs) between January 2015 and December 2021 were included in the study. All patients with leptomeningeal seeding were excluded from our study, as this condition is linked to a poorer prognosis [13]. Pertinent clinical information, including patient characteristics and radiological findings, was collected and recorded in a computerized database (SPSS, version 25, IBM Corp., Armonk, NY). The Karnofsky Performance Score (KPS) was utilized to assess patients based on their preoperative neurological functional status [14–16].

Defined target volumes were manually assessed according to specified magnetic resonance imaging (MRI) sequences using commercially available software (TumorTracking Tool, IntelliSpace Portal 5.0, Philips, Best, the Netherlands) as previously described [17]. Perifocal edema volume and 4th ventricle volume were assessed in T2-weighted MRI, tumor volume and necrosis volume were measured in post-contrast T1-weighted MRI.

Perilesional edema was categorized aligned to existing literature into three groups: perifocal, unilateral, or bilateral. Perifocal edema was characterized as edema extending less than 5 mm from the tumor. Edema spreading more than 5 mm from the tumor without crossing the midline was labelled as unilateral, and edema crossing the midline was designated as bilateral [18–20].

An Evans ratio greater than 0.3 was considered a radiological indicator of hydrocephalus [21]. Additionally, periventricular cerebrospinal fluid (CSF) capping, defined as symmetric hyperdense signals, particularly in the frontal horns on T2-weighted MRI scans obtained preoperatively, was analyzed as a potential risk factor for hydrocephalus [22].

Oncological treatment decisions were reached during the weekly tumor board meetings at the neuro-oncological center of the University Hospital Bonn [23].

The study was conducted in accordance with the Declaration of Helsinki, and the protocol was approved by the Ethics Committee of the University Hospital Bonn (No. 250/19). Informed consent was not sought as a retrospective study design was employed.

### Surgical procedure and definition of postoperative hydrocephalus

As per institutional protocol, the semi-sitting position represents our standard position for resection of posterior fossa tumors and was used for all patients in this study, with intraoperative monitoring via transesophageal echocardiography conducted by an anesthesiologist [24]. Prior to resective surgery, a prophylactic EVD was routinely placed in all patients to manage potential postoperative hydrocephalus. EVD placement was performed through either the Kocher or Fraizer point, depending on the surgeon’s preference.

During the first three postoperative days, the EVD was connected to a drainage system positioned 15 cm above the external auditory meatus and was only opened if the intracranial pressure (ICP) exceeded 15 cmH2O. This three-day protocol accounts for the surgery-related edema, which typically peaks within 24 h. If daily CSF drainage was less than 50 ml per day over the first three days, the EVD was removed at the end of day three. In cases where drainage exceeded 50 ml per day, an EVD weaning trial was conducted on the fourth postoperative day. During this trial, the drainage system was clamped for 24 h. Postoperatively, if clinical deterioration occurred, particularly signs and symptoms of hydrocephalus, and/or cranial CT imaging 24 h after EVD closure showed signs of hydrocephalus, a diagnosis of permanent hydrocephalus was made, indicating the need for ventriculoperitoneal (VP) shunt implantation. Transient postoperative hydrocephalus was defined as a successful EVD weaning trial, leading to EVD removal at the conclusion of the weaning trial on the fourth postoperative day.

### Statistical analysis

Statistical analysis was conducted with IBM^®^ SPSS^®^ Statistics (version 27, IBM Corp., Armonk, NY) and R software environment (version 4.4.0; R Foundation, Vienna, Austria). Descriptive statistics were used to present quantitative data as means with standard deviations (SD), while categorical variables were expressed as counts and percentages. Univariate analysis involved Fisher’s exact test for nominal data and the chi-squared test for multinomial data. Multivariable analysis was conducted using logistic regression and ANOVA.

Statistical significance was determined using the Bonferroni correction, in which the original p-value was divided by the number of variables tested. In this study, a p-value of < 0.05/19 = 0.0026 was considered significant.

ROC Curves were generated using IBM^®^ SPSS^®^ Statistics (version 27, IBM Corp., Armonk, NY) and then the optimal cut-offs with the highest Youden Index (sensitivity + specificity − 1) were selected. The threshold was defined in such a way that sensitivity was considered more important than specificity, i.e. the relative cost of of a false negative classification (as compared with a false positive classification) was set to 10.

## Results

### Baseline characteristics

Between 2015 and 2021, 130 patients were surgically treated for PFMs at the authors’ neuro-oncology center. All patients received EVD implantation. Median age at time of surgery was 64 (IQR 57–71) years. Forty-five patients (35%) had a preoperative KPI < 70. Sixty-seven of 130 patients with PFMs (52%) suffered from lung cancer, followed by breast cancer (17%) and gastrointestinal-tract cancer (15%). Sixty patients (46%) exhibited multiple intracranial BMs. Among them, 37 patients (28%) had PFMs exclusively in the infratentorial region, while the remaining patients exhibited PFMs in both the supra- and infratentorial locations. All surgeries were performed in the semi-sitting position.

Postoperative hydrocephalus occurred in 14 of 130 patients (11%). Among these 14 patients, 8 patients (57%) experienced transient hydrocephalus, while 6 patients (43%) experienced permanent hydrocephalus, necessitating subsequent implantation of a ventriculo-peritoneal shunt system. For further details on patient and tumor characteristics, see Table 1.

**Table 1:** Baseline characteristics *

Total (*n* = 130)	No patients (%)
**Sex**	
Male	62 (48)
Female	68 (52)
**Median age** (IQR, yrs)	64 (57–71)
**Karnofsky index**	
≥ 70	85 (65)
< 70	45 (35)
**Tumor of primary site**	
Lung	67 (51)
Breast	22 (17)
Gastrointestinal tract	20 (15)
Melanoma	3 (2)
Renal	2 (2)
Prostate	2 (2)
Other/CUP	14 (11)
**Multiple BMs**	60 (46)
**Median OS (95% CI**,** months)**	11.2 (5.9–16.8)
**Postoperative hydrocephalus**	14 (11)
Transient	8 (57)
Permanent	6 (43)

* Values represent number of patients unless otherwise indicated (%).

ASA, American society of anesthesiology; BM, brain metastasis; CI, confidence interval; CUP, cancer of unknown primary; IQR, interquartile range; min. minutes; No, number; OS, overall survival; yrs, years.

We analyzed our cohort for intra- and postoperative adverse events typically associated with the semi-sitting position. Two patients (1%) experienced pulmonary embolism (PE), and six (5%) developed postoperative pneumonia. The occurrence of these complications did not significantly differ between patients with a reduced functional status, as indicated by a KPS score < 70 (Table 2).

**Table 2:** Postoperative complications in patients with KPS score ≥ 70 and KPS score < 70*

Total (n = 130)	Patients with KPS score ≥ 70 n = 85 (65%)	Patients with KPS score < 70 n = 45 (35%)	p-value
PE	1 (1)	1 (2)	0.93
pneumonia	4 (5)	2 (4)	0.89
ICU-stay after surgery > 14-days	4 (5)	6 (13)	0.76

* Values represent number of patients unless otherwise indicated (%)

ICU, Intensive care unit; KPS, Karnofsky Performance Status; PE, pulmonary embolism

Furthermore, we evaluated the group of patients who underwent postoperative shunt implantation, focusing on the risk of shunt occlusion potentially caused by tumor cell seeding. None of the patients with permanent hydrocephalus who received a shunt experienced occlusion. In the 14 patients who developed postoperative hydrocephalus, there was no radiological evidence of intraventricular hemorrhage. None of the patients in our cohort developed latent or late-onset hydrocephalus during the 30-day postoperative period.

### Patient- and disease-related characteristics dependent on the occurrence of postoperative hydrocephalus

Patients with postoperative hydrocephalus exhibited a median edema-to-tumor-volume ratio of 1.1 (IQR 0.5–2.7) compared to 2.6 (IQR 1.3–3.9) in patients without postoperative hydrocephalus (*p* < 0.00005). Additionally, median 4th ventricle to tumor ratio in patients with postoperative hydrocephalus was significantly lower than in patients without postoperative hydrocephalus (0.02 (IQR 0.01–0.03) versus 0.09 (IQR 0.03–0.34), *p* < 0.00005). In ten of 14 patients with postoperative hydrocephalus (71%), the preoperative MRI showed direct imaging-morphological contact of the PFM with the 4th ventricle. In contrast, this was observed in only 43 of 116 patients without postoperative hydrocephalus (37%, *p* = 0.001). Patients with postoperative hydrocephalus significantly more often exhibited multiple BMs compared to patients without postoperative hydrocephalus (71% versus 43%, *p* = 0.001) (Table 3).

**Table 3:** Patient- and disease-related characteristics dependent on the occurrence of postoperative hydrocephalus *

Total (*n* = 130)			Patients with postoperative HC *n* = 14 (%)	Patients w/o postoperative HC *n* = 116 (%)	*p*-value
**Median age** (IQR, yrs)			60 (51–68)	64 (57–72)	0.04
**Female sex**			8 (57)	60 (52)	0.04
**Karnofsky index < 70**			5 (36)	57 (49)	0.04
**Multiple BMs**			10 (71)	50 (43)	**0.001**
**Tumor of primary site**					0.04
	Lung		11 (79)	56 (48)	
	Breast		1 (7)	21 (18)	
	Gastrointestinal		1 (7)	19 (17)	
	Others		1 (7)	20 (17)	
**Preoperative radiological findings**					
	Evans ratio (> 0.3)		10 (71)	53 (46)	0.005
	CSF Capping		9 (64)	43 (37)	0.004
	Edema classification				0.01
		Perilesional (< 0.5 ml)	4 (29)	48 (41)	
		Unilateral (> 0.5 ml)	2 (14)	31 (27)	
		Bilateral	8 (57)	37 (32)	
	Contact with 4th ventricle		10 (71)	43 (37)	**0.001**
	Median tumor volume (IQR, cm^3^)		20 (11-29.2)	16.7 (7.7–24.9)	0.02
	Median edema volume (IQR, cm^3^)		14(6.8–22)	10.2 (3.3–16.7)	0.05
	Median necrosis volume (IQR, cm^3^)		4.2 (1.5–7.7)	4 (1.7–8.1)	0.05
	Median 4th ventricle volume (IQR, cm^3^)		0.35 (0.33–0.37)	0.5 (0.42–0.68)	0.02
	Median 4th ventricle/tumor ratio (IQR)		0.02 (0.01–0.03)	0.09 (0.03–0.34)	**< 0.00005**
	Median edema/tumor ratio (IQR)		1.1 (0.5–2.7)	2.6 (1.3–3.9)	**< 0.00005**
	Median necrosis/tumor ratio (IQR)		0.22 (0.13–0.32)	0.21 (0.09–0.35)	0.01
	Median 4th ventricle/edema ratio (IQR)		0.25 (0.11–0.39)	0.23 (0.09–0.8)	0.03
	Median 4th ventricle/edema and tumor ratio (IQR)		0.02 (0.009–0.031)	0.03 (0.01–0.05)	0.01
**EVD insertion point**					0.03
	Kocher		13 (93)	99 (85)	
	Fraizer		1 (7)	17 (15)	

* Values represent number of patients unless otherwise indicated (%)

ASA, American society of anesthesiology; BM, brain metastasis; CI, confidence interval; CSF, cerebrospinal fluid; EVD, external ventricular drainage; HC, hydrocephalus; IQR, interquartile range; KPS, Karnofsky Performance Score; min. minutes; No. number; OS, overall survival; w/o, without; yrs. years

Median tumor volume (*p* = 0.02), peritumor edema volume (*p* = 0.05), central necrosis volume (*p* = 0.05) and 4th ventricle volume (*p* = 0.02) did not significantly correlate to the occurrence of postoperative hydrocephalus following resection of PFMs.

### Multivariable regression analysis identifies risk factors for postoperative hydrocephalus occurrence

**Fig. 1:**
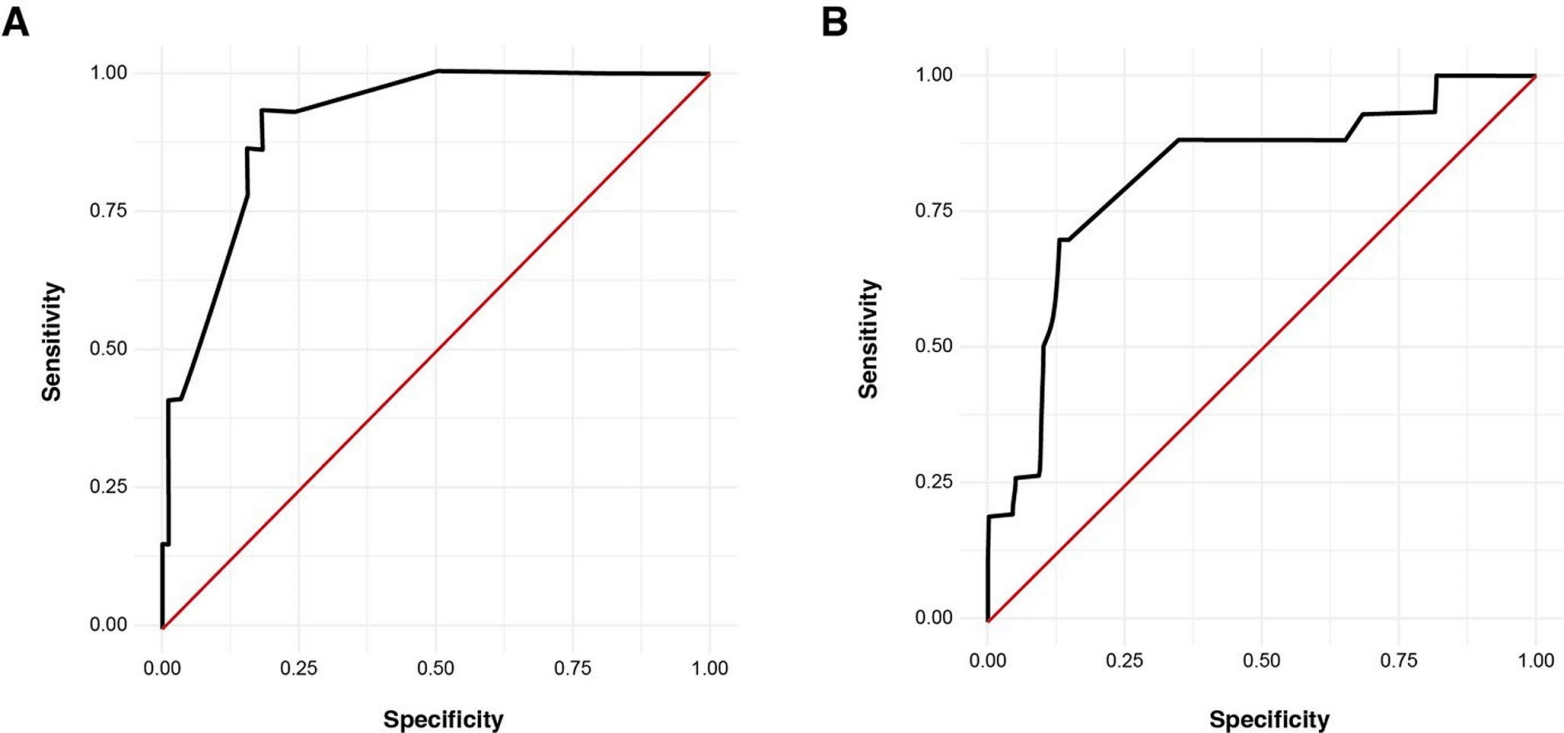
ROC-analysis demonstrating the cut off values for (**A**) the 4th ventricle-to-tumor-volume ratio (≤ 0.02, sensitivity 92% and specificity 81%) and (**B**) the edema-to-tumor-volume ratio (≤ 0.85, sensitivity 71.4% and specificity 89.7%)

ROC-analysis revealed cut off values for 4th ventricle-to-tumor ratio and edema/tumor ratio as ≤ 0.02 (sensitivity 92% and specificity 81%) and ≤ 0.85 (sensitivity 71.4% and specificity 89.7%) to significantly discriminate between the occurrence of postoperative hydrocephalus following surgery for PFMs (Fig. 1).

Based on these cut-off values, we conducted a multivariable regression analysis to identify preoperatively detectable predictors of postoperative hydrocephalus. The analysis revealed a 4th ventricle-to-tumor-volume ratio ≤ 0.02 (OR 33.1, 95% CI 3.8-284.3, *p* = 0.001), an edema to tumor volume ratio ≤ 0.85 (OR 10.6, 95% CI 2.4–47.4, *p* = 0.002), an imaging-morphological contact to the 4th ventricle (OR 5, 95% CI 1.4–18, *p* = 0.013), and multiple BMs (OR 2.4, 95% CI 1-5.9, *p* = 0.045) as preoperatively assessable risk factors for transient and permanent hydrocephalus following resection of PFMs (Table 4).

**Table 4:** Multivariable regression analysis identifies risk factors for postoperative hydrocephalus occurrence *

Total (n = 130)	Patients with postoperative HC (n = 14)	Patients w/o postoperative HC (n = 116)	p-value	OR	95% CI
Multiple BMs	10 (8)	50 (39)	0.045	2.4	1-5.9
Contact with 4th ventricle	10 (8)	43 (33)	0.013	5	1.4–18
4th ventricle/tumor ratio	0.02 ± 0.01	0.09 ± 0.25	0.001	33.1	3.8-284.3
Edema/tumor ratio	1.1 ± 1.6	1.3 ± 2.6	0.002	10.6	2.4–47.4

* Values represent number of patients unless otherwise indicated (%)

BM, brain metastasis; CI, confidence interval; HC, hydrocephalus; n, number; OR, odds ratio; w/o, without

**Fig. 2:**
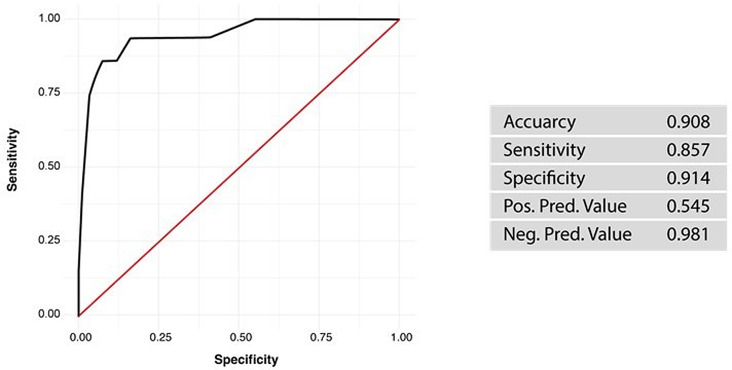
ROC-analysis evaluating the statistical predictive value of the risk factors identified for postoperative hydrocephalus

**Fig. 3:**
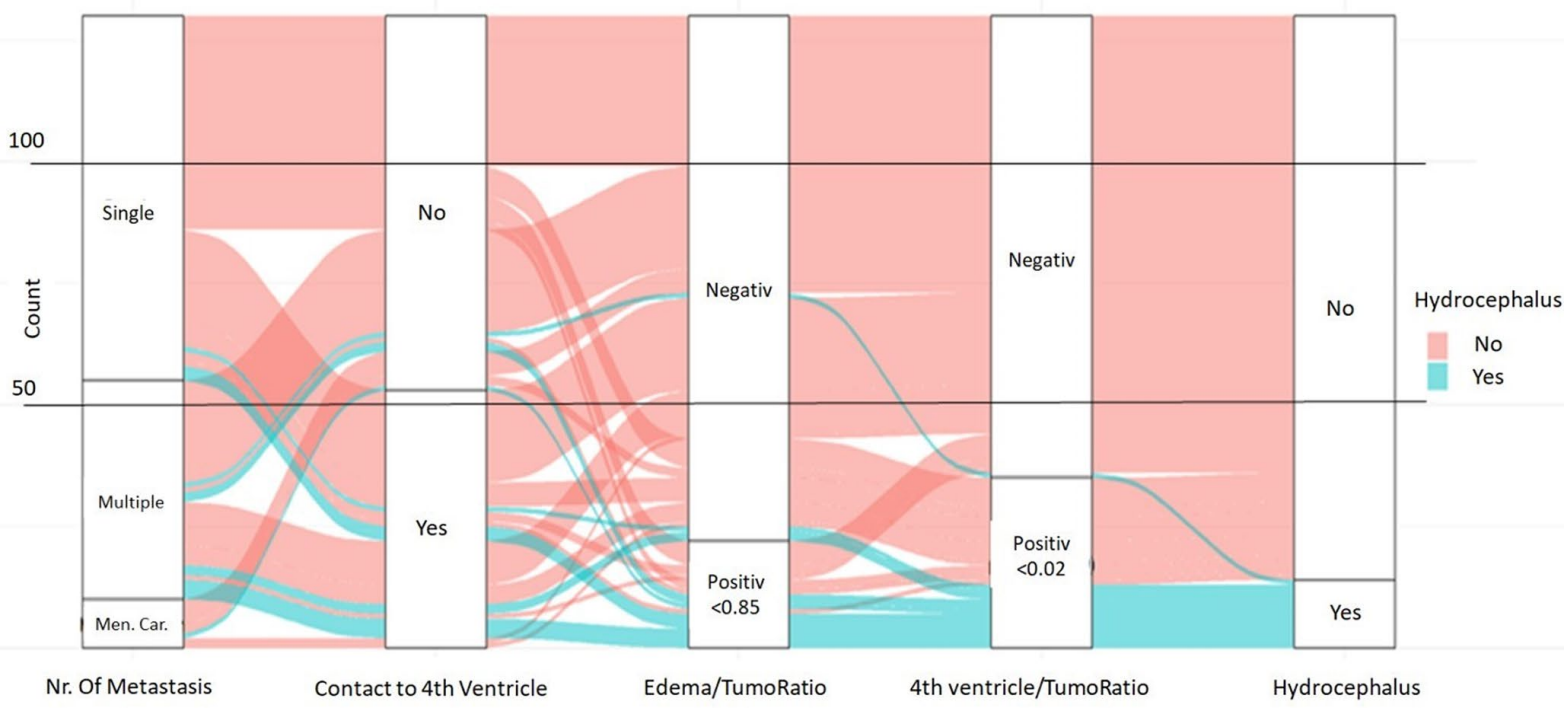
Alluvial plot of identified risk factors for postoperative hydrocephalus occurrence

The combined predictive value of these four variables was further analyzed employing a multiple logistic regression model and a corresponding ROC analysis, revealing an accuracy of 91%, a sensitivity of 86% and a specificity of 91% (Figs. 2 and 3).

## Discussion

Our findings showed few complications related to the semi-sitting position; this aligns with complication rates reported in other studies [25]. However, the complication rate did not significantly differ between patients with high and low KPS scores, supporting our standard approach of routinely using this position regardless of preoperative functional status of the patient.

This present study identifies preoperatively detectable risk factors for the occurrence of postoperative hydrocephalus following surgery for PFMs.

These factors include multiple intracranial brain metastases (BMs), which may exert additional compressive effects in the posterior fossa, a confined space that leads to the consumption of volumetric soft reserves, such as the ventricular system, potentially causing hydrocephalus. Radiological tumor contact to 4th ventricle as a risk factor for developing postoperative hydrocephalus, which align with existing literature [20]. A likely explanation is that resecting tumors in close proximity to the 4th ventricle often requires opening the ventricle, which may increase the risk of iatrogenic hydrocephalus due to the introduction of hemorrhagic sediment, tumor cells, or other debris into the CSF. A fourth ventricle-to-tumor volume ratio of ≤ 0.02 suggests a large tumor volume relative to a small fourth ventricle, making it more sensitive to any pathological volumetric changes in the posterior fossa. Similarly, an edema-to-tumor volume ratio of ≤ 0.85 indicates a relatively larger solid tumor compared to perilesional edema. This may reflect the temporary nature of perilesional edema, which is often reversible after tumor resection. The combined mass of the solid tumor and surrounding edema affects CSF circulation, with larger masses presenting a higher risk for hydrocephalus development. However, distinguishing whether the tumor or edema primarily contributes to the mass effect is crucial. For example, a large mass with a small solid tumor and extensive edema may have less of an obstructive effect than a similarly sized mass where the solid tumor is proportionally larger. Additionally, the degree of intraoperative retraction needed to resect the tumor’s solid part may influence CSF circulation; tumors with smaller solid components require less retraction.

Recognizing these risk factors may aid in guiding the preoperative decision-making for prophylactic EVD placement.

The effective treatment or prevention of postoperative hydrocephalus in patients undergoing resective surgeries of PFMs is a topic of ongoing debate [7, 10, 11]. EVD placement prior to or during resection of PFMs has often been employed as a preventive measure against postoperative hydrocephalus or intracranial hypertension. This practice could help reduce the chances of herniation and fatal outcomes [18, 20, 26, 27] as well as long-term complications of hydrocephalus [20, 28–31]. However, the decision of prophylactic EVD placement is often influenced by the surgeon’s personal preference rather than by clinical evidence [18, 20]. Furthermore, the procedure is not universally endorsed due to the associated risks of complications, such as upward cerebellar herniation, hemorrhaging in the supratentorial or infratentorial regions and potential persistence of hydrocephalus [28, 31–49].

Postoperative persistent hydrocephalus occurs in around 10–40% of pediatric patients and approximately 5–8% of the adult population [31, 40, 50]. In our study, 11% of patients developed postoperative hydrocephalus, slightly exceeding previous literature. This may be attributed to the inclusion of transient hydrocephalus and the less restrictive criteria used in our study, where postoperative hydrocephalus cases with a successful EVD weaning trial in the first few postoperative days. In accordance to several previous studies [18, 40], patients´ characteristics (age, sex, BMI, ASA score etc.) did not have a significant effect on hydrocephalus development in this present analysis.

Numerous studies have attempted to pinpoint predictive factors for postoperative hydrocephalus regardless of tumor entity such as young age (< 3 years) [32, 51, 52], presence of moderate to severe symptomatic hydrocephalus at the time of diagnosis [9, 18, 20, 27, 34, 37, 38, 46, 47, 50, 52–62], transependymal edema, an Evan’s index exceeding 0.4, occurrence of pseudomeningocele and CSF leakage, as well as wound infection and incomplete tumor resection [9, 18, 20, 27, 34, 37, 38, 46, 47, 50, 52–62].

However, a significant limitation of previous studies is the heterogeneity of tumor entities included in these analyses. In this context, the present study is the first to specifically focus on PFMs. We identified four risk factors for the development of postoperative transient hydrocephalus that can be effectively assessed through preoperative routine imaging in clinical practice. Using a multiple logistic regression model and a corresponding ROC analysis, combination of these variables yielded a sensitivity of 85.7% and specificity of 91.4%. This proposed model may provide valuable guidance in prospectively individual risk-based decision-making on EVD placement. Further validation in larger multicenter cohorts will define its true role and applicability in clinical practice.

## Limitations

Our study has several constraints that should be considered when interpreting the results. Firstly, it is based on retrospective data, which may introduce certain biases inherent to such studies. Additionally, following the ten-in-one rule for multivariate analysis, ideally only one predictor should be identified for every ten events analyzed. In our study, however, four predictors were identified for a relatively small number of postoperative hydrocephalus cases, which limit the robustness of these findings. All Analysis were carried out on the whole data set, without dividing it into training and test data, as the sample size was considered too small. Consequently, the results are likely overfitted to the data and cannot be generalized. Despite these limitations, our results may serve as a step towards the creation of multicenter registries aimed at enhancing clinical decision-making processes.

## Conclusion

The present study identifies preoperatively collectable risk factors for the development of postoperative hydrocephalus following surgery for PFMs. These risk factors include multiple intracranial BMs, radiological contact with the 4th ventricle, an edema-to-tumor volume ratio of ≤ 0.85, and a fourth ventricle-to-tumor volume ratio of ≤ 0.02. Recognizing these factors through routine preoperative imaging may help guiding the decision-making process regarding the need for prophylactic EVD placement. Further validation in larger, multicenter studies is necessary to confirm the predictive power and broad applicability in clinical practice.

## Data Availability

No datasets were generated or analysed during the current study.

## Declarations

### Previous presentations

Data included in this manuscript have been presented in part at the 75^h^ national day of Deutsche Gesellschaft für Neurochirurgie/German Society of Neurosurgery (DGNC) in Göttingen 06/2024 and sections day of Sektionstagung Deutsche Gesellschaft für Neurochirurgie/German Society of Neurosurgery (DGNC) 09/2024.

## References

[1] Villano JL et al (2015) Incidence of brain metastasis at initial presentation of lung cancer. Neuro Oncol 17(1):122–128 24891450 10.1093/neuonc/nou099 PMC4483041

[2] Davis FG et al (2012) Toward determining the lifetime occurrence of metastatic brain tumors estimated from 2007 United States cancer incidence data. Neuro Oncol 14(9):1171–1177 22898372 10.1093/neuonc/nos152 PMC3424213

[3] Ghia A et al (2007) Distribution of brain metastases in relation to the hippocampus: implications for neurocognitive functional preservation. Int J Radiat Oncol Biol Phys 68(4):971–977 17446005 10.1016/j.ijrobp.2007.02.016

[4] Delattre JY et al (1988) Distribution of brain metastases. Arch Neurol 45(7):741–744 3390029 10.1001/archneur.1988.00520310047016

[5] Mahajan A et al (2017) Post-operative stereotactic radiosurgery versus observation for completely resected brain metastases: a single-centre, randomised, controlled, phase 3 trial. Lancet Oncol 18(8):1040–1048 28687375 10.1016/S1470-2045(17)30414-X PMC5560102

[6] Albright L, Reigel DH (1977) Management of hydrocephalus secondary to posterior fossa tumors. J Neurosurg 46(1):52–55 830815 10.3171/jns.1977.46.1.0052

[7] Taylor WA, Todd NV, Leighton SE (1992) CSF drainage in patients with posterior fossa tumours. Acta Neurochir (Wien) 117(1–2):1–6 1514423 10.1007/BF01400627

[8] Shalit MN, Ben Ari Y, Eynan N (1979) The management of obstructive hydrocephalus by the use of external continuous ventricular drainage. Acta Neurochir (Wien) 47(3–4):161–172 474210 10.1007/BF01406401

[9] Schmid UD, Seiler RW (1986) Management of obstructive hydrocephalus secondary to posterior fossa tumors by steroids and subcutaneous ventricular catheter reservoir. J Neurosurg 65(5):649–653 3772453 10.3171/jns.1986.65.5.0649

[10] Rappaport ZH, Shalit MN (1989) Perioperative external ventricular drainage in obstructive hydrocephalus secondary to infratentorial brain tumours. Acta Neurochir (Wien) 96(3–4):118–121 2711895 10.1007/BF01456169

[11] Papo I, Caruselli G, Luongo A (1982) External ventricular drainage in the management of posterior fossa tumors in children and adolescents. Neurosurgery 10(1):13–15 7057970

[12] Papo I, Caruselli G, Luongo A (1981) CSF withdrawal for the treatment of intracranial hypertension in acute head injuries. Acta Neurochir (Wien) 56(3–4):191–199 7270257 10.1007/BF01407230

[13] Hawkins A et al (2024) Top ten Tips Palliative Care clinicians should know about caring for people with Leptomeningeal Disease. J Palliat Med 10.1089/jpm.2024.0291 39315927

[14] Hamed M et al (2021) Preoperative metastatic brain Tumor-Associated Intracerebral Hemorrhage is Associated with Dismal Prognosis. Front Oncol 11:699860 34595109 10.3389/fonc.2021.699860 PMC8476918

[15] Schuss P et al (2021) The impact of prolonged mechanical ventilation on overall survival in patients with surgically treated brain metastases. Front Oncol 11:658949 33816316 10.3389/fonc.2021.658949 PMC8013703

[16] Schneider M et al (2020) Comorbidity Burden and Presence of multiple intracranial lesions are Associated with adverse events after Surgical Treatment of patients with brain metastases. Cancers (Basel), 12(11) 10.3390/cancers12113209 PMC7692304 33142701

[17] Bahna M et al (2022) Tumor-associated epilepsy in patients with brain metastases: necrosis-to-tumor ratio forecasts postoperative seizure freedom. Neurosurg Rev 45(1):545–551 33988803 10.1007/s10143-021-01560-y PMC8827395

[18] Won SY et al (2019) A novel grading system for the prediction of the need for cerebrospinal fluid drainage following posterior fossa tumor surgery. J Neurosurg 132(1):296–305 30611134 10.3171/2018.8.JNS181005

[19] Won SY et al (2020) A novel grading system for the prediction of the need for cerebrospinal fluid drainage following posterior fossa tumor surgery. J Neurosurg 132(1):296–305 30611134 10.3171/2018.8.JNS181005

[20] Won SY et al (2020) Management of hydrocephalus after resection of posterior fossa lesions in pediatric and adult patients-predictors for development of hydrocephalus. Neurosurg Rev 43(4):1143–1150 31286305 10.1007/s10143-019-01139-8

[21] Evans WA Jr. (1942) An encephalographic ratio for estimating ventricular enlargement and cerebral atrophy. Archives Neurol Psychiatry 47(6):931–937

[22] Neema M et al (2009) Normal findings on brain fluid-attenuated inversion recovery MR images at 3T. AJNR Am J Neuroradiol 30(5):911–916 19369605 10.3174/ajnr.A1514 PMC3003332

[23] Schäfer N et al (2021) Implementation, relevance, and virtual adaptation of neuro-oncological tumor boards during the COVID-19 pandemic: a nationwide provider survey. J Neurooncol 153(3):479–485 34115248 10.1007/s11060-021-03784-w PMC8192684

[24] Shabo E et al (2023) Asymptomatic postoperative cerebral venous sinus thrombosis after posterior Fossa Tumor surgery: incidence, risk factors, and Therapeutic options. Neurosurgery 92(6):1171–1176 36728332 10.1227/neu.0000000000002340

[25] Hurth H et al (2024) The risk of intraoperative venous air embolism from neurosurgical procedures performed in the lounging position: an in-depth analysis of detection, management, and outcomes of 1000 consecutive cases. J Neurosurg,: p. 1–11 10.3171/2024.5.JNS232449 39303312

[26] Ghani E et al (2003) Role of cerebrospinal fluid diversion in posterior fossa tumor surgery. J Coll Physicians Surg Pak 13(6):333–336 12814531

[27] Imieliński BL et al (1998) Posterior fossa tumors in children–indications for ventricular drainage and for V-P shunting. Childs Nerv Syst 14(6):227–229 9694333 10.1007/s003810050217

[28] Ruggiero C et al (2004) Endoscopic third ventriculostomy in the treatment of hydrocephalus in posterior fossa tumors in children. Childs Nerv Syst 20(11–12):828–833 15221247 10.1007/s00381-004-0938-y

[29] Di Rocco F et al (2013) Endoscopic third ventriculostomy and posterior fossa tumors. World Neurosurg 79(2 Suppl):S18e15–S18e19 10.1016/j.wneu.2012.02.018 22381845

[30] Ji W et al (2013) [Management of obstructive hydrocephalus before posterior fossa tumor resection in children]. Nan Fang Yi Ke Da Xue Xue Bao 33(11):1696–1698 24273282

[31] Dewan MC et al (2017) The durability of endoscopic third ventriculostomy and ventriculoperitoneal shunts in children with hydrocephalus following posterior fossa tumor resection: a systematic review and time-to-failure analysis. J Neurosurg Pediatr 19(5):578–584 28291428 10.3171/2017.1.PEDS16536

[32] Due-Tønnessen BJ, Helseth E (2007) Management of hydrocephalus in children with posterior fossa tumors: role of tumor surgery. Pediatr Neurosurg 43(2):92–96 17337918 10.1159/000098379

[33] El-Gaidi MA, El-Nasr AH, Eissa EM (2015) Infratentorial complications following preresection CSF diversion in children with posterior fossa tumors. J Neurosurg Pediatr 15(1):4–11 25380176 10.3171/2014.8.PEDS14146

[34] Gaskill SJ, Marlin AE (1999) Posterior fossa tumors in children: indications for ventricular drainage and for VP shunting. Childs Nerv Syst 15(4):147–148 10361963 10.1007/s003810050356

[35] Fritsch MJ et al (2005) Hydrocephalus in children with posterior fossa tumors: role of endoscopic third ventriculostomy. J Neurosurg 103(1 Suppl):40–42 16122003 10.3171/ped.2005.103.1.0040

[36] Sainte-Rose C et al (2001) Management of hydrocephalus in pediatric patients with posterior fossa tumors: the role of endoscopic third ventriculostomy. J Neurosurg 95(5):791–797 11702869 10.3171/jns.2001.95.5.0791

[37] Helmbold LJ et al (2019) Predictive factors associated with ventriculoperitoneal shunting after posterior fossa tumor surgery in children. Childs Nerv Syst 35(5):779–788 30929070 10.1007/s00381-019-04136-w

[38] de Santos R et al (2008) Hydrocephalus in posterior fossa tumors in children. Are there factors that determine a need for permanent cerebrospinal fluid diversion? Childs Nerv Syst 24(12):1397–1403 18516610 10.1007/s00381-008-0649-x

[39] Taheri SA, Wani MA, Lewko J (1989) External ventricular drainage and passive vs. active neurosurgical intervention in the management of hypertensive intracerebral hemorrhage with rupture into the ventricles. J Neurosurg Anesthesiol 1(3):233–240 15815279 10.1097/00008506-198909000-00005

[40] Marx S et al (2018) Frequency and treatment of hydrocephalus prior to and after posterior fossa tumor surgery in adult patients. Acta Neurochir (Wien) 160(5):1063–1071 29455408 10.1007/s00701-018-3496-x

[41] Zuccarello M, Dollo C, Carollo C (1985) Spontaneous intratumoral hemorrhage after ventriculoperitoneal shunting. Neurosurgery 16(2):245–246 3974838 10.1227/00006123-198502000-00024

[42] Waga S et al (1981) Intratumoral hemorrhage after a ventriculoperitoneal shunting procedure. Neurosurgery 9(3):249–252 7301065

[43] Santhanam R, Balasubramaniam A, Chandramouli BA (2009) Fatal intratumoral hemorrhage in posterior fossa tumors following ventriculoperitoneal shunt. J Clin Neurosci 16(1):135–137 19013806 10.1016/j.jocn.2008.02.016

[44] Ostling L, Raffel C (2015) Complications following preresection shunting in patients with posterior fossa tumors. J Neurosurg Pediatr 15(1):1–3 25380172 10.3171/2014.9.PEDS14440

[45] Epstein F, Murali R (1978) Pediatric posterior fossa tumors: hazards of the preoperative shunt. Neurosurgery 3(3):348–350 740134 10.1227/00006123-197811000-00003

[46] Culley DJ et al (1994) An analysis of factors determining the need for ventriculoperitoneal shunts after posterior fossa tumor surgery in children. Neurosurgery, 34(3): pp. 402-7; discussion 407-8 10.1227/00006123-199403000-00003 8190214

[47] Gnanalingham KK et al (2003) The natural history of ventriculomegaly and tonsillar herniation in children with posterior fossa tumours–an MRI study. Pediatr Neurosurg 39(5):246–253 14512688 10.1159/000072869

[48] Moscardini-Martelli J et al (2021) Upward transtentorial herniation: a new role for endoscopic third ventriculostomy. Surg Neurol Int 12:334 34345475 10.25259/SNI_140_2021 PMC8326076

[49] Cuneo RA et al (1979) Upward transtentorial herniation: seven cases and a literature review. Arch Neurol 36(10):618–623 485890 10.1001/archneur.1979.00500460052006

[50] Tamburrini G et al (2008) Endoscopic third ventriculostomy: the best option in the treatment of persistent hydrocephalus after posterior cranial fossa tumour removal? Childs Nerv Syst 24(12):1405–1412 18813936 10.1007/s00381-008-0699-0

[51] Srinivasan HL et al (2020) Does pre-resection endoscopic third ventriculostomy prevent the need for post-resection CSF diversion after pediatric posterior fossa tumor excision? A historical cohort study and review of the literature. J Neurosurg Pediatr 25(6):615–624 32084638 10.3171/2019.12.PEDS19539

[52] Riva-Cambrin J et al (2009) Predicting postresection hydrocephalus in pediatric patients with posterior fossa tumors. J Neurosurg Pediatr 3(5):378–385 19409016 10.3171/2009.1.PEDS08298

[53] Schijman E et al (2004) Management of hydrocephalus in posterior fossa tumors: how, what, when? Childs Nerv Syst 20(3):192–194 14762680 10.1007/s00381-003-0900-4

[54] Gopalakrishnan CV et al (2012) Factors predicting the need for cerebrospinal fluid diversion following posterior fossa tumor surgery in children. Pediatr Neurosurg 48(2):93–101 23038047 10.1159/000343009

[55] Foreman P et al (2013) Validation and modification of a predictive model of postresection hydrocephalus in pediatric patients with posterior fossa tumors. J Neurosurg Pediatr 12(3):220–226 23808727 10.3171/2013.5.PEDS1371

[56] Bateman GA, Fiorentino M (2016) Childhood hydrocephalus secondary to posterior fossa tumor is both an intra- and extraaxial process. J Neurosurg Pediatr 18(1):21–28 27035552 10.3171/2016.1.PEDS15676

[57] Bognár L et al (2003) Analysis of CSF shunting procedure requirement in children with posterior fossa tumors. Childs Nerv Syst 19(5–6):332–336 12709823 10.1007/s00381-003-0745-x

[58] Dias MS, Albright AL (1989) *Management of hydrocephalus complicating childhood posterior fossa tumors.* Pediatr Neurosci, 15(6): pp. 283-9; discussion 290 10.1159/000120484 2489586

[59] Kumar V et al (1996) Ventriculo-peritoneal shunt requirement in children with posterior fossa tumours: an 11-year audit. Br J Neurosurg 10(5):467–470 8922705 10.1080/02688699647096

[60] Lee M et al (1994) Management of hydrocephalus in children with medulloblastoma: prognostic factors for shunting. Pediatr Neurosurg 20(4):240–247 8043462 10.1159/000120797

[61] Lin CT, Riva-Cambrin JK (2015) Management of posterior fossa tumors and hydrocephalus in children: a review. Childs Nerv Syst 31(10):1781–1789 26351230 10.1007/s00381-015-2781-8

[62] Srinivasan HL et al (2020) Does pre-resection endoscopic third ventriculostomy prevent the need for post-resection CSF diversion after pediatric posterior fossa tumor excision? A historical cohort study and review of the literature. J Neurosurg Pediatr,: p. 1–10 10.3171/2019.12.PEDS19539 32084638

